# A gating mechanism for border node assisted association of wireless personal area networks

**DOI:** 10.1186/2193-1801-1-12

**Published:** 2012-08-16

**Authors:** Saima Zafar, Ali Hammad Akbar, Sana Jabbar

**Affiliations:** 1 Department of Electrical Engineering, University of Engineering &Technology, UET, Lahore, Pakistan; 2 Al-Khwarzmi Institute of Computer Science, University of Engineering & Technology, Lahore, Pakistan

**Keywords:** Gating, Personal area networks, Border nodes, Diffusion

## Abstract

IEEE 802.15.4 based Wireless Personal Area Networks (WPANs) are envisioned to play a vital role in the application centric ubiquitous networks. In IEEE802.15.4 networks, the use of non-interfering logical channels in multiple PANs, operating in a Personal Operating Space (POS) ensures to control the scope of PAN-directed broadcasts, reduce received energy per PAN device, and minimize interference at the physical layer. At the downside the inter-PAN communication is not possible with this setup as PANs in the same region remain unaware of each other’s presence. This situation hinders sharing information of common interest amongst PANs and accessing infrastructure networks hopping through multiple PANs. In this paper, we present a comprehensive architecture enables neighboring PANs to communicate by diffusing into each other through border nodes. The basic idea is to time share logical channels to perform a gating operation by border nodes. Our contribution includes detection of border nodes, discovery of neighboring PAN(s), a common channel based gating protocol, interest diffusion and inter-PAN data transfer. We mathematically analyze the proposed architecture and carry out simulations in Network Simulator (ns2) for performance evaluation. Our simulations show that the proposed architecture gives optimal performance when multiple border nodes are able to perform gating.

**Electronic supplementary material:**

The online version of this article (doi:10.1186/2193-1801-1-12) contains supplementary material, which is available to authorized users.

## Introduction

IEEE802.15.4 Low-Rate Wireless Personal Area Networks (LR-WPAN) are envisioned to support ubiquitous computing like wireless sensor networks. The standard defines the physical (PHY) layer and medium access control (MAC) layer specifications for low power devices that wirelessly communicate within Personal Operating Space (POS) around 10 meters or less at low data rates. WPANs are expected to find a very important role in the application centric ubiquitous networks. With the emergence of this technology, newer application scenarios of WPANs are also emerging. Body Area Networks (BANs) and Car Area Networks (CANs) are just the snapshots of what applications might evolve into the future. Whatsoever the diversification of these networks, one thing might be said with certainty about them—these networks would need to collaborate at various levels and degrees. Although the collaboration within a PAN is a widely studied area, little work can be found for collaboration across PANs. The focus of this work is inter-WPAN communication.

In IEEE802.15.4 WPANs (IEEE802.15.4-[2003]), while a single logical channel may be used for multiple PANs in the same POS each with a unique PAN-Id, the usage of a non-interfering logical channel for each PAN is highly desirable. It ensures to contain PAN-directed broadcasts, reduces receiving energy per PAN device, and minimizes interference at the physical layer. However, it also inhibits inter-PAN communication. The PANs in the same POS remain unaware of each other’s presence and form isolated islands. This situation adversely affects the likelihood to exploit the synergistic role of PANs, e.g., sharing information of common interest amongst PANs and accessing infrastructure networks (e.g., the Internet) hopping through multiple PANs.

In this paper, we take up the problem of allowing communication between PANs in the same POS that are operating in different logical channels. We present an architecture extending an earlier proposal ([Zafar et al. 2010]) that systematically allows neighboring PANs to communicate with each other by diffusing into each other. The diffusion takes place through gating operation performed by nodes that reside at the border of the two non-interfering PANs. Specifically, our contributions include a) border nodes detection algorithm, b) neighbor PAN discovery, c) A common channel based gating mechanism, d) interest advertisement and e) inter-PAN data transfer. The remainder of the paper is organized as follows. In section 2, we discuss the related work. Section 3 presents the proposed architecture in detail. In section 4 we mathematically analyze the proposed architecture and in section 5 we present experimental results based on ns2 simulations. Finally; section 6 summarizes results and concludes the paper.

### Related work

Broadly speaking, the contribution in this work is two-fold, namely border nodes identification and cluster diffusion. Hence the related work is classified in two sections; border nodes identification methods and the current state of the art in cluster merging and cluster diffusion.

Different boundary nodes identification methods are surveyed in ([Khan et al. 2008]; [Zhang et al. 2009]; [Mallery & Medidi 2008]; [Wang et al. 2006]; [Ahmed et al. 2005]). In ([Khan et al. 2008]) Khan et al. presents a survey of boundary detection algorithms for wireless sensor networks. They categorize the boundary detection algorithms into three types, namely the geometrical approaches, the statistical approaches and topological approaches. In ([Zhang et al. 2009]) Zhang et al. propose two innovative algorithms for individual sensor nodes to detect whether they are located on the coverage boundary, i.e., the boundary of a coverage hole or network separation. Their algorithms are founded on two new computational geometric techniques called localized Voronoi and neighbor embracing polygons. Mallery and Medidi in ([Mallery & Medidi 2008]) highlight the importance of boundary detection in sensor networks by reasoning that the ability to geometrically signify sensed phenomena within a sensor network can offer extra concise view as compared to details of all nodes detecting a phenomenon. Wang et al. propose an algorithm in ([Wang et al. 2006]) which is founded on constructing a shortest path tree by flooding the network. They utilize the Nearest Common Ancestors (NCA) method to find holes in the network. In ([Ahmed et al. 2005]) this algorithm is further improved. The principal behind this algorithm is creating iso-contours on the basis of hop count distance from the root node.

Cluster-merging has been investigated in ([Jung et al. 2007]; [Willig et al. 2010]; [Jurdak et al. 2008]; [Bandara et al. 2008a]; [Ferreira & Rocha 2007a]; [Wei et al. 2006]; [Campos et al. 2005]; [Misic et al. 2005]; [Misic & Fung 2007]). Wei et al. ([Wei et al. 2006]) describe a cluster-merging algorithm where link Optimization gets the priority. In 802.15.4 networks, a very close work to ours has been done by Misic et al. ([Misic et al. 2005]; [Misic & Fung 2007]) where IEEE802.15.4 network clusters are interconnected through a master–slave bridge. However, they take up the issue of cluster merging as a post-neighboring PAN detection process, bypassing the definition of the mechanics to discover a neighboring PAN. They propose inter-PAN communication in beacon-enabled mode. According to their model, inter-PAN communication is established through a single point, viz. the PAN coordinator.

### Motivation

**Figure 1:**
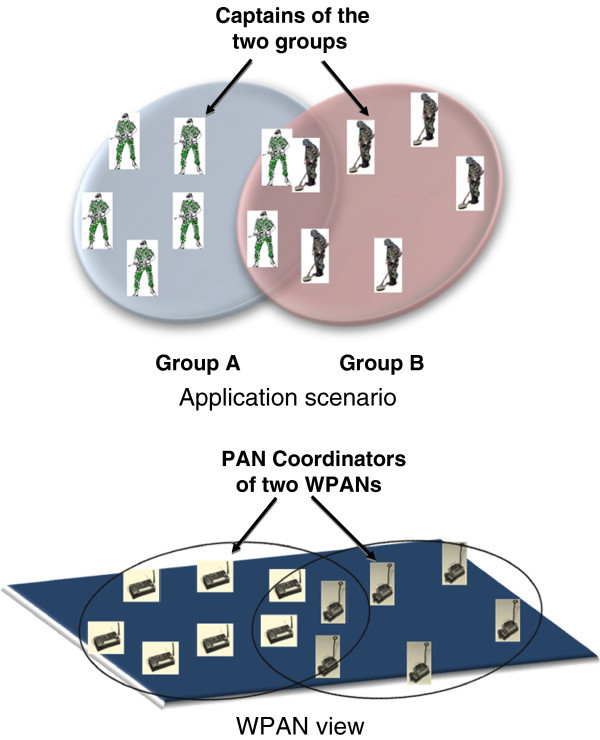
**Military application scenario for PAN diffusion through border nodes of neighboring WPANs.**

The following scenario is aimed at signifying the need to diffuse WPANs. Consider a military application as shown in Figure 1 which shows troops belonging to different corps who work in their respective groups, and also corroborate closely with each other by sharing tactical information. The troops in each company (group of upto 150 people) are each equipped with a sensor device. For example company ‘A’ forms PAN A and company ‘B’ forms PAN B. As their mission, troops in the company ‘A’ of the *corps of engineers* detect man and tank mines and share this information with their allies in company ‘B’ of the *corps of infantry* using wireless networks as the communication medium. This information is diffused across the two PANs through sensor devices that are in close proximity of each other. Likewise, troops in the infantry role can share information regarding the time and location of next offensive with their allies in the engineering role. In this scenario each company is required to keep its identity intact thus mandating PAN diffusion.

### Bridge

The BordeR-node assIsted Diffusion through Gating mEchanism (BRIDGE) architecture comprises of the following basic procedures:

- Border nodes identification (BAIT)
- Gating mechanism (BIND)
- Inter-PAN data communication (SEND)

**Figure 2:**
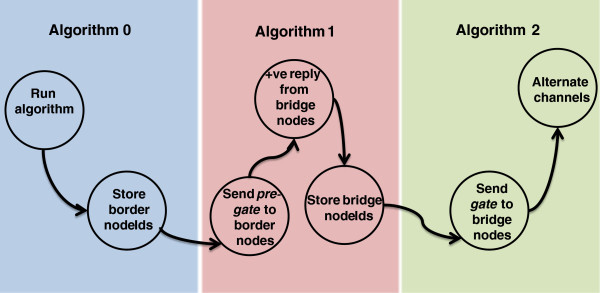
**Procedural flow and functions of BRIDGE.**

The procedural flow of BRIDGE with functional description of procedures is shown in Figure 2.

### Bordering nodes indentificAtIon for gaTing (BAIT)

BAIT operation is initiated at the PAN coordinator where border nodes are detected and their Ids are stored at PAN initialization. Node Id assignment in WPAN follows formula, where *FC* stands for ‘address of first child node’, *MC* stands for ‘maximum allowed children’, *AP* stands for ‘address of parent node’ and *N* represents the ‘*Nth* child node’. PAN coordinator runs Algorithm 0 (Additional file 1) for border node detection.

Node Ids of border nodes are stored at PAN coordinator, at this stage PAN coordinator sends pre-gate command to border nodes to initiate foreign PAN(s) discovery. Border nodes run algorithm 1 (Additional file 2) and respond to pre-gate command. Response is positive if foreign PAN is discovered and negative otherwise.

**Figure 3:**
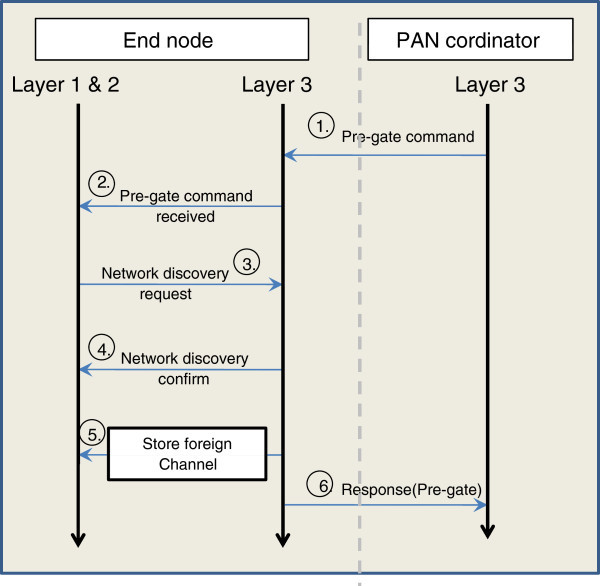
**Timing diagram for pre-gate.**

PAN coordinator stores border nodes Ids that positively respond and also retrieves foreign PAN information on the basis of foreign PAN Id. Those border nodes that positively respond to *pre-gate* are termed as bridge nodes. Timing diagram is given in Figure 3.

### Border Nodes for GatINg through Duty cycle assignment (BIND)

The BIND procedure is carried out through Algorithm 2 (Additional file 3). The *gate* command is issued by the PAN coordinator upon receiving the affirmative acknowledgement [Resp(*pre-gate*) = true] from border node(s) and executed at the border nodes. The PAN coordinator issues duty cycle and resumes normal operation.

**Figure 4:**
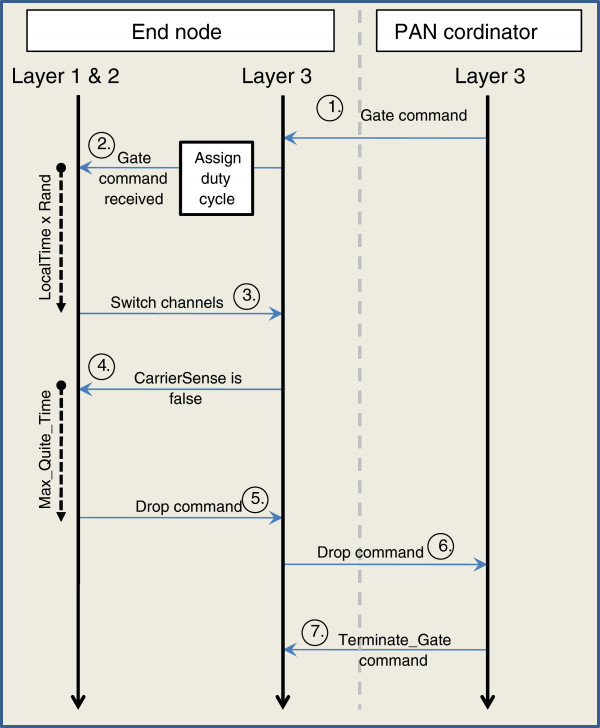
**Execution of*gate*command.**

The border node(s) execute the *gate* command, alternating between the local channel and the foreign channel based on duty cycle issued by the PAN coordinator (Figure 4). Duty Cycle is the ratio of time elapsed in neighbor PAN logical channel and parent PAN logical channel. The node randomizes the start of switching between the local and foreign channel. If bridge node does not overhear foreign PAN i.e. *CarrierSense* is false for time (*MAX_QUITE_Time*) it sends DROP message to PAN coordinator. Upon receiving this message from all bridge nodes, PAN coordinator sends *Terminate_Gate* command to all bridge nodes.

### Border SEnsor Node based inter-PAN Data transfer (SEND)

When a neighboring PAN is discovered, coordinator advertises about the presence of the foreign PAN to all sensor nodes. Before data transfer, sensor nodes must use an interest-based mechanism to solicit data. The data structure for such interest is application-specific and does not mandate explanation here. There are three possible ways for inter PAN interest and data transfer.

- Paradigm 1: The home-PAN notifies all sensor nodes under its control about the presence of foreign PAN with a specific role. An ordinary sensor node in PAN ‘A’ if interested in information of interest from foreign PAN forwards inter-PAN data to the coordinator of PAN ‘A’. PAN ‘A’ coordinator has information about bridge nodes therefore it sends this data to PAN ‘B’ coordinator through bridge nodes. PAN ‘B’ coordinator then sends data to the destination sensor node of PAN ‘B’.
- Paradigm 2: Another option is for the home-PAN to broadcast to all sensor nodes, not only information about foreign-PANs but also the Ids of bridge nodes through which foreign-PANs can be accessed. In this way, sensor nodes in PAN ‘A’ may send data directly to bridge nodes. For this way of communication, coordinator must announce the Ids of bridge nodes along which type of foreign PAN to which a node can connect through some particular bridge nodes. The bridge node then sends data to the destination node in PAN ‘B’.
- Paradigm 3: An additional merged mode of data transfer may also be employed where inter-PAN data transfer is mostly through PAN coordinator. But if bridge node is one hop away from the sending sensor node the sensor node directly sends data to bridge node. In this way those sender nodes that are located near bridge nodes do not have to access bridge nodes through PAN coordinator which can be at a large hop-count away from the sender. This mixed mode also requires notification of bridge node Ids along with foreign-PAN information to all sensor nodes of the network.

### Mathematical analysis

This section presents a simple mathematical model for estimating total delay in sending data from a sender node N_s_ in PAN A to receiver node N_r_ in PAN B using BRIDGE protocol, the ‘Duty Cycle’ estimation for bridge node and the effect of data rates and multiple bridge nodes on network performance and BRIDGE protocol performance. The analysis provides an insight into the behavior of BRIDGE protocol. The model draws on the concepts introduced in ([Bandara et al. 2008b]; [Ferreira & Rocha 2007b]).

### Network model for analyzing BRIDGE architecture

The important factors that impact throughput and delay in BRIDGE are number of bridge nodes, data transfer paradigm, network topology, transmission range of nodes, network traffic pattern and the behavior of MAC protocol. The impact of some of these factors is analyzed in this section. The list of notations that are used in the analysis is given in Table 1.

**Table 1:** **List of notations for mathematical analysis of BRIDGE protocol**

Notation	Description	Notation	Description
*T*_*total*_	Delay from N_s_ in PAN A to N_r_ in PAN B	*T*_*qo*_	Queuing delay at ordinary node
*T*_*1*_	Delay from N_s_ to PAN coordinator A	*T*_*q*_*(av)*	Av. queuing delay at bridge node
*T*_*PA*_	Total processing delay at PAN coordinator A	*T*_*qb*_	Queuing delay at bridge node
*T*_*2*_	Delay from PAN coordinator A to connect node	*Λ*	Data arrival rate at a node
*T*_*b*_	Total delay at bridge node	*M*	Data processing rate at a node
*T*_*switch*_	Delay at bridge node due to switching WPANs	*N*_*b*_	Number of packets requested by PAN B
*T*_*3*_	Total delay from bridge node to N_r_ in WPAN B	*T*_*A*_	Time at bridge node in home PAN A
*h*_*1*_	Hop count from N_s_ to PAN coordinator A	*T*_*B*_	Time at bridge node in PAN B
*h*_*2*_	Hop count from PAN coordinator A to bridge node	*D*_*r*_	Inter-WPAN data at bridge node
*P*_*a*_	Probability that N_s_ has data to send	*D*_*i*_	Intra-WPAN data at bridge node
*T*_*s*_	Transmission time for successful transmission	*λ*_*1*_	Arrival rate of sensed data at a node
*T*_*c*_	Time for unsuccessful transmission of packet	*λ*_*2*_	Arrival rate of data to be relayed
*E[a]*	Av. # of attempts for successful packet transmit	*N*_*i*_	Number of neighbors of node _i_
*P*_*s*_	Probability of successful transmission b/w nodes	*P*_*ij*_	Probability data from node *j* is for *i*
*P*_*tr*_	Probability of transmission attempt	*λ*_*B*_	Data arrival rate at a bridge node
*m*	Max unsuccessful attempts →packet is dropped	*λ*_*t*_	Data rate from PAN A to PAN B
*M*	No. of neighbors of nodes transferring data	*b*	Number of border (bridge) nodes

The following assumptions are made for the analysis:

1. The propagation delay is negligible and hence ignored.
2. The terms border node and bridge node are used interchangeably although bridge nodes are those border nodes which discover foreign WPAN and perform gating.

### Delay estimation

Total time *T*_*total*_ that a packet takes to reach from sender node *N*_*s*_ in PAN A to receiver node *N*_*r*_ in PAN B is given by:1

In Eq. (1) *T*_1_ is the delay for the packet to reach from *N*_*s*_ to PAN coordinator in PAN A (PAN coordinator A), *T*_*PA*_ is the total processing delay at PAN coordinator A, *T*_2_is the delay to reach from PAN coordinator A to bridge node, *T*_*b*_ is the total delay at bridge node which includes the processing delay and the delay due to switching from one PAN to another (*T*_*switch*_) and *T*_*3*_ is the total delay to reach from bridge node to N_r_ in PAN B. The delay (*T*_1_) depends upon the number of intermediate nodes between *N*_*s*_ and PAN coordinator A. Delay at each node, including *N*_*s*_ comprises of processing delay, queuing delay, transmission delay and propagation delay ([Kurose & Ross 2007]).2

The delay *T*_*prop*_ is small and therefore negligible. If *h*_1_ is the hop count (number of intermediate nodes) from N_s_ to PAN coordinator A, then *T*_1_ is given as: . MAC protocol in WPAN is CSMA-CA therefore *T*_1_ is variable and depends upon the number of attempts made before channel is accessed. If *P*_α_ is the probability of sender *N*_*s*_ having data to send, then average number of nodes trying to access channel is *P*_α_^*m^. The transmission delay for the packet *T*_*trans*_ is given as:3

where *T*_*c*_ is the time taken by the unsuccessful attempts and *T*_*s*_ is the time taken for successful transmission of packet. E[a] is the average number of attempts required for successful transmission of the packet and is estimated as:4

In Eq. (4), *m* is the maximum number of unsuccessful channel access attempts after which packet is dropped and *P*_*s*_ is the probability of successful transmission between two nodes estimated as:5

In Eq. (5), *M* is the number of neighbors of the two nodes. When network becomes dense, the average number of attempts for successful transmission increases. Probability of successful transmission in time slot (*t*) is *P*_*s*_*P*_*tr*_ such that *P*_*tr*_ is the probability of transmission (attempt).6

*T*_*queue*_ is represented as follows. For an ordinary node (node other than bridge node) in PAN A or PAN B, queuing delay denoted by *T*_*qo*_ is given as:7

Queuing delay in Eq. (7) is for M/D/1 queuing system as given in ([Ferreira & Rocha 2007b]), where  , *λ* is the data arrival rate and *μ* is the data processing rate. When PAN A and PAN B come in same POS and are connected through BRIDGE protocol, *T*_*queue*_ increases due to increase in data traffic. But this increase is no more than if WPANs were connected in a large PAN. The bridge (formed through bridge node) acts like a router in a big PAN with the difference that it can deliver data to only one PAN at a time. BRIDGE operation affects the queuing behavior of bridge node. We propose to divide queue of bridge node in two queues; one for inter-PAN and other for intra-PAN communication. Average queuing delay at bridge node for inter-PAN communication is given as:8

Average queuing delay at bridge node for inter-PAN communication depends on the number of data packets requested by PAN B and the capacity of buffer, given as:9

where *N*_*b*_ are the number of packets requested by PAN B. Additional processing delay at PAN coordinator A is contributed by: (1) delay in border nodes detection, (2) delay in sending *gate* command to bridge nodes and (3) delay in calculating the duty cycle. The delays (1) and (2) are one-time delays. These processing delays at PAN coordinator A constitute *T*_*PA*_.

*T*_*switch*_; the delay at bridge node due to switching from one PAN to another can be estimated as: When data is to be sent from PAN A to PAN B, and bridge node is in PAN B, there is a delay before data is sent to bridge node. This delay is equal to time when bridge node switches back to PAN A. Even if the bridge node is in PAN A, there is some delay as the PAN coordinator has to check whether the remaining time is enough for data to reach the bridge node. If duty cycle is 50% (initially when bridge is formed) then *T*_*A*_ (the time for which bridge node is in its parent PAN i.e. PAN A) and *T*_*B*_ (the time for which bridge node is in neighbor PAN i.e. PAN B) are equal and are given as:10

If PAN A has to send data to bridge node at time *T’*_*A*_ the PAN coordinator has to check the condition:11

where12

In Eq. (12) *h*_*2*_ is the number of hops between PAN coordinator A and bridge node. If given condition is satisfied, PAN coordinator A starts transmission immediately and therefore  Otherwise, PAN coordinator A waits for the next duty cycle and in this case . *T*_*3*_, the total delay from bridge node to *N*_*r*_ includes all the processing, transmission and queuing delays in PAN B and is mainly a function of number of hops (intermediate nodes) from bridge node to PAN coordinator B of PAN B and the number of hops from PAN coordinator B to *N*_*r*_.

### Duty-cycle computation

Duty cycle is calculated based on the amount inter-PAN data and the amount of intra-PAN data. If *D*_r_ data is requested by neighboring PAN and *D*_i_ data for intra-PAN communication i.e., home PAN, duty cycle is calculated as:13

whereand *T*_*A*_ and *T*_*B*_ are as defined earlier i.e., the time for which bridge node is in its parent PAN i.e. PAN A and the time for which bridge node is in neighbor PAN i.e. PAN B. f more than one bridge node exists then duty cycle can be settled in such a way that at least one bridge node is present in each PAN.

### Performance impact of data rates and multiple paths

In multi hop networks there can be two sources of arrival at a node: sensed data and data forwarded by other nodes. If *λ*_1_ is the rate of arrival of sensed data at a node and *λ*_1_ is the rate at which other nodes are sending data at a node which is to be forwarded total data arrival rate at a node *i* is . If *N*_*i*_ is the number of neighbors of node *i* and *P*_ij_ is the probability that the data from node *j* is destined for node *i*, the rate of arrival of data at node *i* which is to be forwarded is given as:14

where . If λ_*i*_>*μ*_*i*_ it means the data arrival rate is greater than data processing rate. In this case buffer will be filled and packets will start dropping. We consider situation when  that is requested number of data packets by PAN B must be less than or equal to the buffer size. Queue length will increase and delay will also increase at each node thus overall delay will increase. If *b* is the number of bridge nodes and if *λ*_1_ is the total data rate that is to be transferred from PAN A to PAN B, then data arrival rate at each bridge node is:15

This shows that as the number of bridge nodes joining two neighboring PANs increases, data arrival rate at each bridge node decreases resulting in efficient performance.

### Performance evaluation

This section documents the performance evaluation of BRIDGE architecture for inter-PAN communication using ns2 simulations. The operation of gating nodes is simulated, concentrating on their capability to transmit data across the collocated PANs. The simulation setup details are given in Table 2.

**Table 2:** **ns2 simulations setup for BRIDGE**

Parameter	Definition/Value
Standard	IEEE 802.15.4
Number of nodes in PAN A	16
Number of nodes in PAN B	11
Data rate	56 kbps – 256 kbps
Simulation time	200 ms
PAN join time	30 ms
Duty cycle	0.5

### Number of border nodes

For the impact of border nodes two partitions of IEEE802.15.4 network were connected through one up to three border nodes and we noted down the delivery ratio when the number of flows was increased. The delivery ratio (or packet delivery ratio) is defined as the ratio of number of packets received by the receiver to the number of packets transmitted by the sender. Figure 4 shows plot for delivery ratio from one to four flows when single, two and three border nodes are used for PAN diffusion respectively. As shown in this graph, the delivery ratio is 100% and deteriorates for two, three and four flows. It is noticed that instead of linear reduction, the reduction is less which is due to the fact that all flows are not active at the same time. The contention would be more if all flows were active simultaneously. It is also noted that the increase in the number of border nodes increases the availability of bridging nodes for ordinary sensor nodes therefore, for an arbitrary flow delivery ratio is additionally increased as compared to a single border node. The delivery ratio is improved to nearly 78% when two border nodes are used for gating and is improved to about 88% when three border nodes are used when compared with 65% delivery ratio when a single border node is used for gating. The results also show that the probability of successful delivery of a flow is a function of number of border nodes(s) in the source channel. As number of border nodes increases, cluster diffusion starts to mirror PAN merger.

On the other hand, when a greater number of border nodes are involved in PANs connectivity, the two PANs do not remain independent, and ultimately a single broadcast domain is created. As a result the possibility of broadcast storms increases, reducing the energies of individual sensor nodes thus increasing node failures. It is concluded that an optimal number of border nodes need to be selected for gating in order to realize best performance in the interconnection of two or more PANs. That optimal number depends upon the amount of data to be transferred and background traffic.

### Background traffic (inter-PAN)

**Figure 5:**
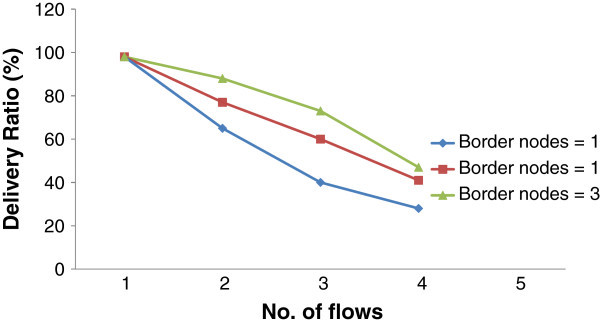
**Delivery ratio vs. no. of flows when a single, two and three border nodes are used for BRIDGE.**

The impact of background traffic can be observed by increasing the number of flows through a single bridge node. As shown in Figure 5, as number of inter-PAN flow increase, latency of data transfer also increases. We transferred a small file from PAN ‘A’ to PAN ‘B’ through a single bridge node and observed the impact of background traffic. As number of flows supported across a bridge node increases latency of data transfer through a bridge node increases. This effect is almost the same for all bridge nodes, therefore if there are more bridge nodes for connecting two neighboring PANs, latency through a single bridge node would be lessened due to the reason that less flows would be supported through a bridge node but as far as the impact of background traffic is concerned, it would be severe at a bridge node with more number of flows as compared to a bridge node with less flows.

### Data delivery paradigms

**Figure 6:**
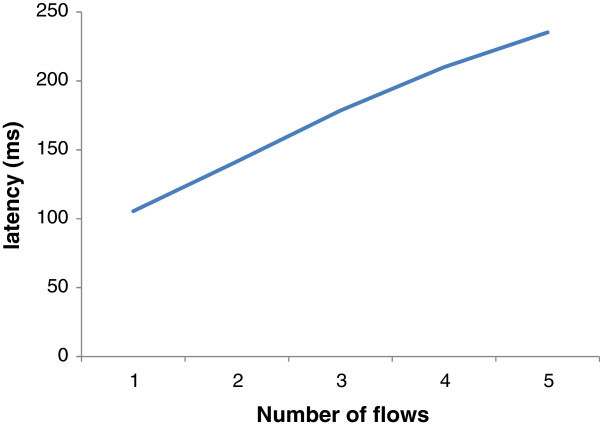
**Impact of background traffic on latency.**

We compared latency as a function of number of flows for data delivery paradigms as defined in BRIDGE design. In paradigm 1, a sending node in PAN ‘A’ sends data to home-PAN coordinator that forwards it to the bridge node to be sent to PAN ‘B’, in paradigm 2, sending node sends data directly to bridge node while in paradigm 3 sending node sends data to PAN coordinator or bridge node on the basis of proximity. In Figure 6 paradigm 2 is the most efficient of the three. This owes to the overhead of route discovery messages in paradigm 3 which otherwise seems to be most efficient. Since paradigm 3 involves route discovery messages for decision about the best path to the bridge node and that contributes to latency therefore it is not the best paradigm as far as latency is concerned. The latency of data transfer in paradigms 1 and 2 depend on proximity of sending node from either PAN coordinator or bridge node which is variable. Depending upon the location of sending node either of the first two paradigms performs well. When the number of inter-PAN data flows is more, paradigm 2 outperforms paradigm 1 because there are more chances for sending nodes to be located far from PAN coordinator and nearer to bridge nodes. This trend is reflected in our simulations as we increase the number of flows.

**Figure 7:**
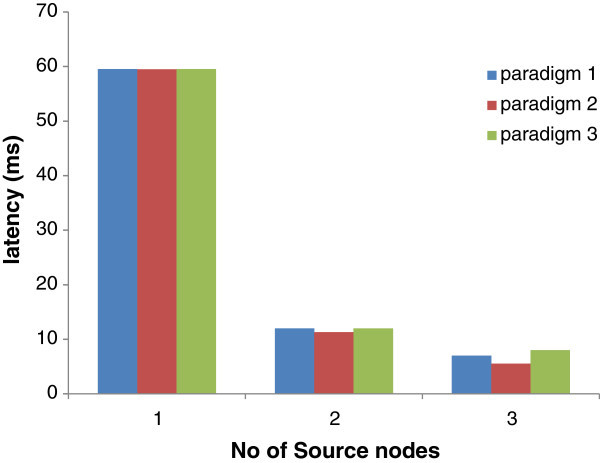
**Impact of data delivery paradigms on latency of data transfer.**

## Conclusion

In IEEE802.15.4 networks, when non-interfering logical channels are used in multiple PANs operating in a Personal Operating Space the inter-PAN communication is not possible because the PANs in the same region remain unaware of each other’s presence. This situation adversely hinders realizing the pervasive and synergistic vision of PANs, e.g., sharing information of common interest amongst PANs and accessing infrastructure networks hopping through multiple PANs. In this paper, we have presented a novel mechanism to allow communication between multiple PANs in the same POS that are using different logical channels. We have proposed a framework that enables neighboring PANs to communicate with each other by diffusing into each other through “bordering nodes”. The simulation results show improvement in delivery ratio when number of border nodes is increased, and also hinting at the likely detrimental effects when the borders nodes are indefinitely increased.

## Electronic supplementary material

Additional file 1: **Algorithm 0:** Border nodes identification {Executed at the PAN Coordinator}. (DOC 24 KB)

Additional file 2: **Algorithm 1:** On receiving pre-gate from PC {Executed by border nodes}. (DOC 28 KB)

Additional file 3: **Algorithm2:***Gate* Command {Issued by the PAN Coordinator on Receiving Resp(*pre-gate*)}. (DOC 23 KB)

## References

[CR1_17] Ahmed N, Kanhere S, Jha S (2005). The holes problem in wireless sensor networks: a survey. ACM Sigmobile Mobile Computing and Communications Review J (MC2R).

[CR2_17] Bandara H, Jayasumana A, Illangasekare T (2008a). Cluster tree based self organization of virtual sensor networks. Proceedings of IEEE Globecom workshop on Wireless Mesh and Sensor Networks.

[CR3_17] Bandara H, Jayasumana A, Illangasekare T (2008b). Cluster tree based self organization of virtual sensor networks. In: Proceedings of IEEE Globecom workshop on Wireless Mesh and Sensor Networks: Paving the Way to the Future or yet Another…?.

[CR4_17] Campos R, Pinho C, Ricardo M, Ruela J, Poyhonen P, Kappler C (2005). Dynamic and Automatic Interworking between Personal Area Networks using Composition. Proceedings of 16th IEEE International Symposium on Personal Indoor and Mobile Radio Communications.

[CR5_17] Ferreira LS, Rocha RM (2007a). Multi-Channel Clustering Algorithm to Improve Performance of WSNs. Proceedings of Conference on Telecommunications.

[CR6_17] Ferreira L, Rocha R (2007b). Multi-Channel Clustering Algorithm to Improve Performance of WSNs. Proceedings of 6th Conference on Telecommunications.

[CR7_17] (2003). “Draft IEEE Standard for Telecommunications and Information Exchange Between Systems - LAN/MAN Specific Requirements - Part 15: Wireless Medium Access Control (MAC) and Physical Layer (PHY) Specifications for Low Rate Wireless Personal Area Networks (WPAN)”.

[CR8_17] Jung S, Chang A, Gerla M (2007). Comparisons of ZigBee Personal Area Network (PAN) Interconnection Methods.

[CR9_17] Jurdak R, Nafaa A, Barbirato A (2008). Large scale environmental monitoring through integration of sensor and mesh networks. Sensors, Special Issue on Wireless Sensor Technologies and Applications, J.

[CR10_17] Khan I, Mokhtar H, Merabti M (2008). A Survey of Boundary Detection Algorithms for Sensor Networks. Annual postgraduate symposium on the convergence of telecommunications, networking and broadcasting.

[CR11_17] Kurose J, Ross K (2007). Computer Networking, A Top-Down Approach Featuring the Internet.

[CR12_17] Mallery CJ, Medidi M (2008). Robust Edge Detection in Wireless Sensor Networks. Proceedings of GLOBECOM, 2008.

[CR13_17] Misic J, Fung CJ (2007). The impact of master–slave bridge access mode on the performance of multi-cluster 802.15.4 network. Computer Networks J.

[CR14_17] Misic J, Fung J, Misic VB (2005). Interconnecting 802.15.4 clusters in master–slave mode: queueing theoretic analysis. Proceedings of 8th International Symposium on Parallel Architectures, Algorithms, and Networks.

[CR15_17] Wang Y, Gao J, Mitchell JSB (2006). Boundary recognition in sensor networks by topological methods. Proceedings of MOBICOM, 2006.

[CR16_17] Wei Z, Hui-Min C, Hao W (2006). Cluster Merging Algorithm with Link Optimization for Wireless Sensor Networks. Proceedings of 2nd International Conference on Wireless Communications, Networking and Mobile Computing.

[CR17_17] Willig A, Karowski N, Hauer J (2010). Passive discovery of IEEE 802.15.4-based body sensor networks. Ad Hoc Networks J.

[CR18_17] Zafar S, Akbar AH, Amjad M, Shams S, Chaudhry SA, Seuk J, Roh B, Kim K (2010). BRIDGE: border-node assistance for common interest-based diffusion through a gating mechanism for collocated WPANs. Proceedings of International Conference on Emerging Technologies.

[CR19_17] Zhang C, Zhang Y, Fang Y (2009). Localized algorithms for coverage boundary detection in wireless sensor networks. Wireless Networks J.

