# Supplementary material for: A gating mechanism for border node assisted association of wireless personal area networks
Source: Springerplus. 2012 Aug 16;1:12. doi: 10.1186/2193-1801-1-12 (PMC3725855; doi:10.1186/2193-1801-1-12)
Supplement: Supplementary file 1 — Additional file 1: Algorithm 0: Border nodes identification {Executed at the PAN Coordinator}. (DOC 24 KB) [file 40064_2012_17_MOESM1_ESM.doc]

**Algorithm 0: Border nodes identification {Executed at the PAN Coordinator}**

If PAN-initialization or node joins/leaves PAN

Border nodes list = {0}//Node Id of PAN coordinator

From neighbor tables of one hop-count nodes from PAN coordinator

If one hop-count nodes scatter uniformly (derived from neighbor tables and MC reached)

Border nodes list = {Node Ids of one hop-count nodes}//Border nodes list updated

Else

Border nodes list = {0, Node Ids of one hop-count nodes}//Border nodes list updated

End

From neighbor tables of two hop-count nodes from PAN coordinator

Border nodes list include {Node Ids of two hop-count nodes & Node Ids of one hop-count nodes without

child nodes}//Border nodes list updated

From neighbor tables of three hop-count nodes from PAN coordinator

Border nodes list include {Node Ids of three hop-count nodes & Node Ids of two hop-count nodes with no

child nodes & Node Ids of one hop-count nodes with no child nodes}//Border nodes list updated

Continue till last hop-count nodes

End
