# Supplementary material for: A gating mechanism for border node assisted association of wireless personal area networks
Source: Springerplus. 2012 Aug 16;1:12. doi: 10.1186/2193-1801-1-12 (PMC3725855; doi:10.1186/2193-1801-1-12)
Supplement: Supplementary file 2 — Additional file 2: Algorithm 1: On receiving pre-gate from PC {Executed by border nodes}. (DOC 28 KB) [file 40064_2012_17_MOESM2_ESM.doc]

**Algorithm 1: On receiving pre-gate from PC {Executed by border nodes}**

N
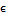
 border nodes

Notify upper layers of receipt of *pre-gate*

Call NLME-NETWORK-DISCOVERY.request //Passive scan over 27 logical channels

Receive NLME-NETWORK-DISCOVERY.confirm

If[(NetworkCount ≠ φ)
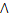
 (PermitJoining = true)]

Store Foreign channel

Set Resp(pre-gate) = true

Send Resp(pre-gate) to PAN coordinator

Else

Set Resp(pre-gate) = false

Switch to CurrentLogicalChannel

End if

Exit
