# Supplementary material for: A gating mechanism for border node assisted association of wireless personal area networks
Source: Springerplus. 2012 Aug 16;1:12. doi: 10.1186/2193-1801-1-12 (PMC3725855; doi:10.1186/2193-1801-1-12)
Supplement: Supplementary file 3 — Additional file 3: Algorithm2:Gate Command {Issued by the PAN Coordinator on Receiving Resp(pre-gate)}. (DOC 23 KB) [file 40064_2012_17_MOESM3_ESM.doc]

**Algorithm2: *Gate* Command {Issued by the PAN Coordinator on Receiving Resp(*pre-gate*)}**

n ⊂ N

1. Notify upper layers of the presence of other PANs in POS

2. Store PAN-IDs of border nodes

3. Send *alternate between LocalChannel and ForeignChannel* command to border nodes

4. Send DS to border nodes

5. Exit
